# Production and purification of immunologically active core protein p24 from HIV-1 fused to ricin toxin B subunit in *E. coli*

**DOI:** 10.1186/1743-422X-6-17

**Published:** 2009-02-06

**Authors:** Alberto J Donayre-Torres, Ernesto Esquivel-Soto, María de Lourdes Gutiérrez-Xicoténcatl, Fernando R Esquivel-Guadarrama, Miguel A Gómez-Lim

**Affiliations:** 1 Centro de Investigación y de Estudios Avanzados (CINVESTAV), Unidad Irapuato, Km 9.6 Libramiento Norte, 36500 Carretera Irapuato-León, Irapuato, Guanajuato, México; 2 Facultad de Medicina, Universidad Autónoma del Estado de Morelos (UAEM), Cuernavaca-Morelos, México; 3 Centro de Investigaciones Sobre Enfermedades Infecciosas, INSP, SSA, Cuernavaca-Morelos, México

## Abstract

**Background:**

Gag protein from HIV-1 is a polyprotein of 55 kDa, which, during viral maturation, is cleaved to release matrix p17, core p24 and nucleocapsid proteins. The p24 antigen contains epitopes that prime helper CD4 T-cells, which have been demonstrated to be protective and it can elicit lymphocyte proliferation. Thus, p24 is likely to be an integral part of any multicomponent HIV vaccine. The availability of an optimal adjuvant and carrier to enhance antiviral responses may accelerate the development of a vaccine candidate against HIV. The aim of this study was to investigate the adjuvant-carrier properties of the B ricin subunit (RTB) when fused to p24.

**Results:**

A fusion between ricin toxin B subunit and p24 HIV (RTB/p24) was expressed in *E. coli*. Affinity chromatography was used for purification of p24 alone and RTB/p24 from cytosolic fractions. Biological activity of RTB/p24 was determined by ELISA and affinity chromatography using the artificial receptor glycoprotein asialofetuin. Both assays have demonstrated that RTB/p24 is able to interact with complex sugars, suggesting that the chimeric protein retains lectin activity. Also, RTB/p24 was demonstrated to be immunologically active in mice. Two weeks after intraperitoneal inoculation with RTB/p24 without an adjuvant, a strong anti-p24 immune response was detected. The levels of the antibodies were comparable to those found in mice immunized with p24 alone in the presence of Freund adjuvant. RTB/p24 inoculated intranasally in mice, also elicited significant immune responses to p24, although the response was not as strong as that obtained in mice immunized with p24 in the presence of the mucosal adjuvant cholera toxin.

**Conclusion:**

In this work, we report the expression in *E. coli *of HIV-1 p24 fused to the subunit B of ricin toxin. The high levels of antibodies obtained after intranasal and intraperitoneal immunization of mice demonstrate the adjuvant-carrier properties of RTB when conjugated to an HIV structural protein. This is the first report in which a eukaryotic toxin produced in *E. coli *is employed as an adjuvant to elicit immune responses to p24 HIV core antigen.

## Background

Gag protein from HIV-1 is a polyprotein of 55 kDa which, during viral maturation, is cleaved to release matrix protein p17, core protein p24 and nucleocapsid protein [1]. Antibodies elicited by the core p24 antigen are an early marker of HIV infection and thereby constitute a major target for HIV diagnosis in early stages of the infection [2]. Antiretroviral drugs can reduce circulating levels of p24 and consequently this antigen can also be used as a marker for evaluating the efficacy of therapy [3,4]. The p24 antigen can elicit lymphocyte proliferation responses, which have been demonstrated to be protective, and it also contains epitopes that prime helper CD4 T-cell responses [5,6]. Thus, p24 is likely to be an integral part of any multicomponent vaccine [7]. Recent trials have suggested that HIV-specific cytotoxic T-lymphocyte activity can be increased in HIV-infected individuals receiving p24 and the antiviral drug zidovudine, reinforcing the possibility for a p24-containing therapeutic vaccine against HIV in the presence of antiretroviral therapy [8].

Ribosome inactivating proteins are a group of cytotoxic proteins. Ricin, the most toxic member of the group, accumulates to high levels in the endosperm of *Ricinus communis *seeds [9]. It is a heterodimeric protein, comprising subunits A and B. Ricin subunit A (RTA) of 31 kDa, is the toxic component of the heterodimer and causes ribosome inactivation, whereas subunit B (RTB) of 34 kDa, is a lectin with galactose-binding properties, responsible for attachment to the surface of target cells [9,10]. Previous experiments had demonstrated that RTB delivers RTA to the cytoplasm of target cells by interacting with glycoproteins and glycolipids located at the cell surface, thereby triggering the endocytic pathway [11,12], via a still unknown receptor [13]. This characteristic has prompted experiments for RTB to be employed as a novel antigen deliverer, leading to the hypothesis that this lectin represents a novel adjuvant-carrier.

On the other hand, the lack of an optimal adjuvant and carrier to enhance antiviral responses has been problematic in vaccine development against HIV [14]. The use of adjuvant-carrier molecules fused to p24 to enhance presentation to the immune system has only been explored on three occasions, employing cholera toxin (CT) subunit B [15], hepatitis B (HB) core antigen [16] and HSP70 from *Mycobacterium tuberculosis *[14]. In this report, we employed a RTB/p24 protein fusion to investigate biological activity *in vitro *and to determine whether the presence of RTB enhances immunological responses to p24 in mice.

## Results

### Construction of RTB/p24 and p24 genes

**Figure 1:**
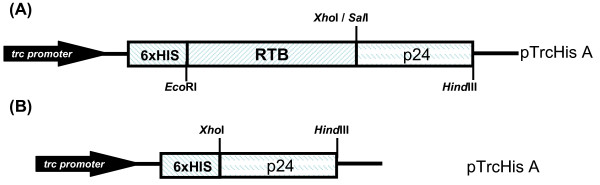
**Expression vectors**. Map showing the constructs employed for *E. coli *expression in vector pTrcHis. (**A**) RTB/p24 and (**B**) p24. The histidine tag is indicated.

The gene fragments p24 and RTB/p24 were cloned by PCR. The core domain p24 was genetically fused to the 3' region of RTB and cloned in a *E. coli *expression vector (Figure 1). The constructs obtained, pTrcHisA-RTB/p24 and pTrcHisA-p24, were subjected to restriction enzyme analysis. The constructs were fully sequenced to confirm in-frame fusion of the two sequences (data not shown). The molecular weight mass of the predicted translation products from sequences RTB/p24 and p24 was 57 kDa and 34 kDa respectively.

### E. coli expression of RTB/p24 and p24 proteins

**Figure 2:**
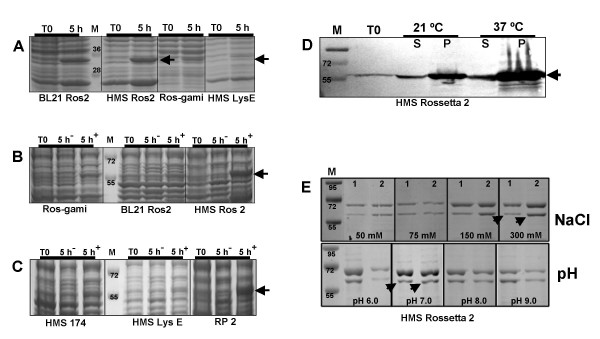
**Expression analysis of chimeric RTB/p24 and p24 in *E. coli***. Total proteins were extracted from *E. coli *cultures, separated by 10% SDS-PAGE and stained with Coomassie. Protein profiles were analyzed after 5 h induction with (5 h^+^) or without IPTG (5 h^-^) and compared with non-induced cultures (T0). Four different *E. coli *strains were tested and these are indicated in the lower part of the panels. Panel A, p24 construct. The arrow indicates the expected band of about 34 kDa. Panels B and C, RTB/p24 construct. The arrows indicate the expected chimeric protein (RTB/p24) of about 57 kDa. The six *E. coli *strains employed are indicated at the bottom of panels. Panel D, western blot of RTB/p24 after expression at two temperatures (21°C and 37°C). We analyzed supernatant (S) and pellet (P) fractions for determination of soluble and insoluble fractions in *E. coli *cultures after 5 h induction. T0 represents non-induced cultures. Western blot was performed using anti-His monoclonal antibody at dilution of 1/1000. Panel E, analysis of different pH and NaCl concentrations on the lysis buffer to improve the solubility of RTB/p24 chimeric protein during affinity chromatography. Lanes 1 and 2 represent 1 ml aliquots of collected fractions. The arrows indicate the expected chimeric protein (RTB/p24). In all panels, M represents molecular weight markers in kDa.

Purification of RTB/p24 and p24 was performed from cell lysates in non-denaturing conditions to keep the proteins in native conformation. In preliminary experiments, we had tested the pellet and supernatant fractions after a 5 h induction, to estimate the solubility of the recombinant proteins. Expression was performed in six *E. coli *strains grown at one of two temperatures (21°C or 37°C). The optimal temperature for obtaining the highest recombinant protein levels for both RTB/p24 and p24 HIV was 37°C. We found that p24 is highly soluble (Figure 2, panel A), whereas RTB/p24 was somewhat insoluble, since we detected high concentration of this protein in the pellet fractions after 5 h induction (Figure 2, panel E). When comparing gene expression in the different *E. coli *strains, p24 was expressed at the highest level in strain HMS Rossetta 2 after 5 h induction (Figure 2, panel A). Interestingly, the same strain was the best expressor for RTB/p24 (Figure 2, panels B and C).

### Purification of E. coli expressed p24 HIV and chimeric RTB/p24 proteins

**Figure 3:**
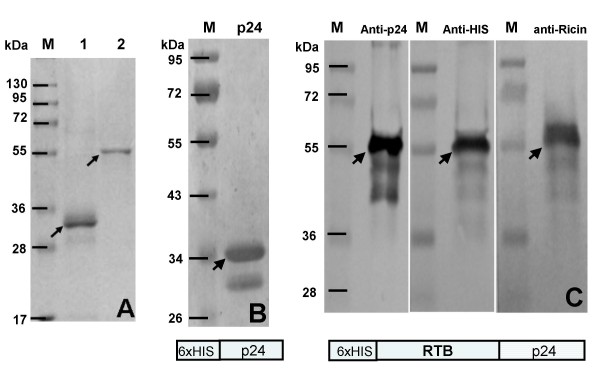
**Purification of recombinant proteins expressed in *E. coli *and immunodetection**. Purification was performed as described in the text. Panel A, purified p24 (Lane 1) and purified RTB/p24 (Lane 2). Arrows indicate the expected proteins. Panel B, western blot of p24 purified protein, immunodetected with anti-p24 antibody at dilution of 1/1000. Panel C, western blot of purified RTB/p24 chimeric protein using three different antibodies, anti-p24 (1/1000), anti-His (1/1000), and anti-Ricin (1/3000). M, molecular weight markers in kDa. At the bottom a diagram of the two constructs is included.

In order to improve solubilization of the *E. coli-*produced-RTB/p24 during affinity chromatography, we tested different NaCl concentrations in combination with different pH until an optimal buffer composition was determined (50 mM TrisHCl, 300 mM NaCl, 20 mM Imidazole, 1 mM PMSF, pH 7.0). Using these conditions, we routinely obtained maximal solubilization of RTB/p24 (Figure 2, panel D) and purification of both proteins was straightforward. The p24 protein, presenting a mw of approximately 34 kDa because of the histidine tag, yields only one band on SDS-PAGE whereas the RTB/p24 fusion, yields a mw of approximately 57 kDa (Figure 3, panel A). Protein concentration was estimated by using a standard curve of bovine serum albumin. Yields of p24 and RTB/p24 recombinant proteins were estimated at 4.6 mg/l and 0.63 mg/l respectively.

### Immunoblot analysis

Analysis by immunoblot of the recombinant purified proteins p24 and RTB/p24 was performed to confirm the identity of the proteins. A band of 34 kDa was detected by specific anti-p24 antibodies although some lower bands were also evident which, we hypothesize, are degradation products. The RTB/p24 fusion was detected by three different antibodies, the monoclonal anti-p24, monoclonal anti-His, and polyclonal anti-ricin antibodies. In all three cases, a band of 57 kDa was detected. The fact that we were able to detect the same band by three different antibodies targeting different components of the chimeric RTB/p24 protein, demonstrate the integrity of the protein after purification (Figure 3, panel C).

### Biological activity of RTB/p24 in vitro

**Figure 4:**
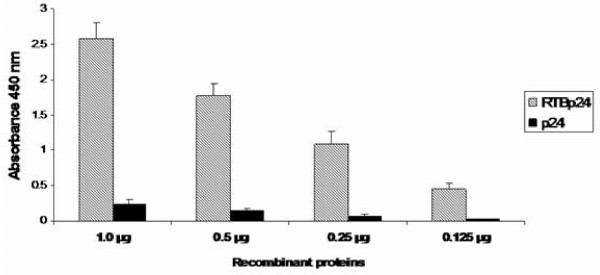
**Functional assay of RTB/p24 chimeric protein**. Capture ELISA was performed using 20 μg of asialofetuin per well. Three concentrations of p24 and RTB/p24 were employed for the assay (1.0 μg, 0.5 μg, 0.25 μg, and 0.125 μg). As primary antibody, monoclonal anti-p24 was used at 1/500 dilution. Samples were done in triplicate and deviations are included.

**Figure 5:**
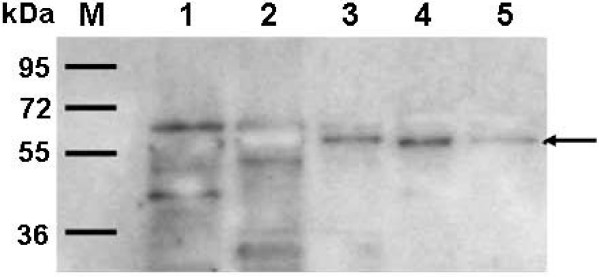
**Analysis by immunodetection of fractions from asialofetuin affinity chromatography**. Five mg of the glycoprotein asialofetuin were immobilized on a 10 ml sepharose column. A total of 25 μg of RTB/p24 purified protein were loaded on to the column. Lane 1, protein not retained by the asialofetuin-sepharose column. Lane 2 column washes. Lane 3 through 5, fractions retained on the column. Immunodetection was performed using the anti-ricin polyclonal antibody at 1/2000 dilution. The arrow indicates the expected protein (57 kDa).

We measured affinity of RTB/p24 to the glycoprotein asialofetuin by capture ELISA. The RTB/p24 fusion showed a strong binding activity to asialofetuin, whereas p24 presented only a residual binding activity which is probably a nonspecific interaction (Figure 4). Our results indicate that the affinity properties of RTB were not altered by fusion to p24. On the contrary, the results suggest that RTB/p24 retains a stable lectin-binding activity with complex sugars. Binding to asialofetuin was further confirmed by affinity column of immobilized asialofetuin. The eluted fractions were tested with anti-ricin antibodies and the 57 kDa band was again detected (Figure 5, lanes 3 to 5). The anti-ricin antibodies also detected other minor bands visible in the non-retained and wash fractions (Figure 5, lanes 1 and 2). Their identity is unknown.

### Immunization of mice with E. coli-based RTB/p24 induced strong immune responses against p24 HIV

**Figure 6:**
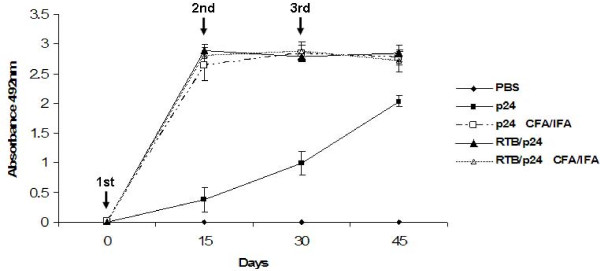
**Levels of IgG antibodies anti-p24 after intraperitoneal immunization**. BALB/c mice were immunized at the days indicated by arrows, with 15 μg of p24 (filled squares) or 30 μg of RTB/p24 in the presence (filled triangles) or absence (open triangles) of CFA/IFA. As negative control mice were inoculated with PBS (filled rhombus). Serum samples were diluted at 1/200, and levels of IgG anti-p24 antibodies were determined by ELISA. All sera samples were tested in triplicate and standard deviations are included.

We were interested to determine whether RTB could function as parenteral and mucosal adjuvant when fused to p24. Groups of female BALB/c mice were inoculated i.p. with varying amounts of the fusion in the presence of complete Freund adjuvant (CFA). The adjuvant was included as control since it is a potent, well-known adjuvant. Two booster immunizations were performed using incomplete Freund adjuvant (IFA) on days 15 and 30 post-priming. Sera were collected before each inoculation and 15 days after the last immunization, and the level of antibodies anti-p24 estimated by ELISA. It was found that RTB/p24 was able to induce high levels of anti-p24 antibodies (Figure 6). The antibody levels, induced in the absence of any adjuvant, were comparable to the levels induced by p24/CFA and RTB/p24 in the presence of CFA/IFA. Core p24 alone did not induce significant levels of antibodies (Figure 6).

**Figure 7:**
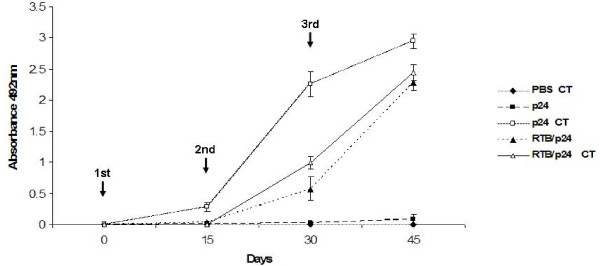
**Levels of anti-p24 antibodies after intranasal immunization with p24 and RTB/p24**. BALB/c mice were intranasally inoculated at the days indicated by arrows with PBS plus 5 μg of CT (filled rhombus), p24 (filled squares), p24 mixed with 5 μg of CT (open squares), RTB/p24 (filled triangles) and RTB/p24 supplemented with 5 μg of CT as adjuvant (open triangles). Serum samples were diluted at 1/200, and levels of IgG anti-p24 were determined by ELISA. Analysis was performed in duplicate and all sera samples were analyzed in triplicate and standard deviations included.

To examine whether RTB could also act as an adjuvant in mucosa, mice were inoculated i.n. following the same schedule and doses as for i.p. immunizations, but using 5 μg of CT, a well-known mucosal adjuvant. Immunization with RTB/p24, p24/CT and RTB/p24/CT induced antibodies with a similar kinetics, reaching the highest levels by day 15 and then remaining stable until the end of the experiment (day 45). Mice immunized with p24 alone also presented detectable levels of anti-p24 antibodies that were increasing with succesive immunizations, albeit at lower levels than in the other treatments (Figure 7). By the day 15, the immune response to p24 alone was about 7-fold lower than in the other immunizations.

## Discussion

The main goal of this work was to express, purify and test the biological activity of a chimeric protein RTB/p24, which could be used to enhance immune responses against HIV-1 p24. RTB, which displays a single peptide monomeric structure, facilitates translocation of molecules into the cell by endocytosis through the cell membrane via uncoated and clathrin-coated vesicles [18]. Both p24 and RTB/p24 were readily produced in *E. coli*, although with different solubility, contrasting the high solubility of the p24 protein with the partial insolubility of the chimeric RTB/p24. Different NaCl concentrations and different pH were tested to improve solubility and eventually it was found that high salt (300 mM) at pH 7 considerably improved solubilization of chimeric RTB/p24. Once purified, RTB/p24 was easily resuspended in PBS. Since proper folding of RTB is an important factor for receptor binding [17,18], non-denaturing conditions were employed throughout purification to keep the proteins in native conformation. Non-glycosylated RTB/p24 showed a strong binding activity to asialofetuin in two different assays, capture ELISA and affinity chromatography. These results suggest that RTB/p24 fusion protein did retain proper conformation in *E. coli *in the absence of glycosylation. Previously, it had been suggested that RTB may retain its activity even when found in a non-glycosylated form and fused to another protein as long as it retains the proper conformation [19]. However, correct conformation of recombinant RTB has not always been achieved. When RTB was fused to the rotavirus antigen NSP4, the fusion protein was denatured during purification and even after renaturation, did it not bind asialofetuin receptors as well as its native counterpart [17].

Our asialofetuin results also suggest that the chimeric protein retained heightened immunogenicity based on the adjuvant properties of the genetically linked RTB. Indeed, the presence of RTB markedly enhanced immune responses in mice to p24 when administered i.p. and the response was comparable to that obtained with p24 and CFA, which is a strong parenteral adjuvant. Similarly, RTB also enhanced immune responses to p24 in mice when administered via the i.n. route. Nevertheless, this response was not as strong as that obtained in mice immunized with p24/CT. This is not surprising since CT is the mucosal adjuvant of choice, however, its use is not allowed in humans because of several side effects, such as olfactory bulb inflammation and severe nasal discharges [20]. Therefore, RTB seems to be a good candidate to avoid these side effects without compromising its immunostimulatory properties. Interestingly, CT did not enhance antibody induction by RTB/p24 when both adjuvants were combined. This was surprising as we had hypothesized that the action of both mucosal adjuvants could be synergistic. Synergy may occur with different mucosal adjuvants, especially if they act via different receptors, but it does not happen in every instance [21]. CT binds GM1-ganglyoside receptors in cell membranes of mammalian cells [22] whereas RTB contains two galactose-binding domains and is able to bind asialo-sugar membrane receptor molecules, including both glycolipids and glycoproteins. [11,12]. Nasal-associated lymphoid tissue contains specialized M cells, which can selectively sample and internalize lectins in the nasal mucosa to be presented to T cells by dendritic cells, macrophages and B cells [23]. As it was shown in this report, the fusion protein RTB/p24 retained lectin activity and therefore, it is likely that after i.n. administration, binding to M cells by RTB increased the uptake and transport of p24 to the nasal lymphoid tissue. On the other hand, we found an enhanced immune response to p24 as early as the second week after the first i.p. immunization with RTB/p24, in comparison to mice immunized with p24 alone, which induced modest anti-p24 antibodies levels at this same timepoint. These results might be explained if administration of the fusion protein RTB/p24 in the peritoneal cavity resulted in its transport to lymph nodes where the fusion could be internalized by antigen presenting cells and presented to T and B cells.

There are some instances in which RTB has been used successfully as a carrier fused to other molecules. For example, RTB has been fused to two cytokines, the granulocyte macrophage colony stimulating factor [24] and interleukin 2 [25] and to the heavy chain of IgG [26]. It has also been fused to the autoantigens proinsulin and glutamic acid decarboxylase [27] and to rotavirus antigens VP7 [28] and NSP4 [17]. The presence of RTB fused to NSP4 resulted in higher levels of serum antibodies, which is consistent with our results, confirming the immunostimulatory function of RTB via i.p. and mucosal routes. The foreign molecule has been usually fused to the N-terminal domain of RTB to avoid steric hindrance by the antigen with RTB galactose receptor binding sites. In this work, we fused p24 to the C-terminal domain of RTB and our results demonstrate that apparently the position of the foreign protein does not affect the carrier-adjuvant abilities of RTB.

Since p24 is weakly immunogenic, adjuvant molecules such as CT subunit B, HB core antigen and HSP70 from *M. tuberculosis *have been employed to enhance presentation of p24 to the immune system. These adjuvants were fused to p24 and immune responses were reported, although with HB core antigen only 90 aa out of 230 aa could be fused to HB core antigen without compromising VLP formation. In all three cases, a strong immune response in mice was reported after i.p. administration, although CFA had to be included with HB core-p24 in order to elicit a response. In our case, fusion to RTB was enough to elicit a strong immune response to p24 via i.p. and intranasal routes, and the levels of antibodies induced were comparable to those induced in the presence of a CFA.

This is the first report in which a eukaryotic toxin produced in *E. coli *is employed as an adjuvant to elicit immune responses to p24 HIV core antigen. The high levels of antibodies obtained after i.n. and i.p. immunization with RTB/p24 should encourage the use of ricin toxin B subunit protein to enhance immune responses against other HIV antigens, with special emphasis in evocating cytotoxic T-lymphocyte responses, which are likely to be an important component of any HIV vaccine candidate.

## Conclusion

We report for the first time the adjuvant properties of *Ricinus communis *toxin B subunit when fused to p24 HIV-1 protein. The chimeric protein was expressed in *E. coli *and purified by affinity chromatography. Yields of p24 and RTB/p24 were estimated to be 4.6 mg/l and 0.63 mg/l respectively. By using the glycoprotein asialofetuin, in capture ELISA and sepharose affinity chromatography assays, we were able to demonstrate binding of the fused protein RTB/p24 to complex sugars, confirming a stable lectin activity. The chimeric protein was able to induce a strong immune response as demonstrated by the mice immunization experiments. Only two weeks after i.p. inoculation with RTB/p24, mice developed a strong anti-p24 antibody response, without the need of an exogenous adjuvant. Intranasal inoculation with RTBp24, triggered levels of anti-p24 antibodies comparable to those obtained by immunization with p24 alone in the presence of cholera toxin. Our results demonstrate that ricin toxin B subunit is an excellent candidate to enhance immunogenicity at the i.p. and i.n. routes of HIV, and probably other, antigens, and potentially to boost cytotoxic T-lymphocyte responses in the context of mucosal protection, a major requirement for a potential HIV vaccine candidate.

## Materials and methods

### Construction of RTB/p24 and p24 genes

Based on the published sequence of proricin (GenBank S40366) [9], we designed primers to amplify RTB using genomic DNA from *R. communis *as template since lectins do not contain introns. The forward (5' CCG CAT GAA TTC ATG GCT GAT GTT TGT ATG GAT CCT GAG CCC ATA 3') and reverse primers (5' ACC TGC CTA TCA CTC GAG AAA TAA TGG TAA CCA TAT TTG GTT 3') incorporated *Eco*RI and *Xho*I sites at the 5' and 3'ends respectively. The DNA coding for HIV-1 p24 was amplified by PCR using a cDNA encoding *gag *as template, kindly provided by Dr. Yong Kang (University of Western Ontario), using a forward (5' GTC GAC CCT ATA GTG CAG AAC 3') and reverse primers (5' AAG CTT TCT AGA TTA TTA CAA AAC TCT TGC TTT ATG 3'), incorporating *Sal*I, *Hind*III and *Xba*I sites. After cloning the PCR products in the Topo 2.1 vector, p24 was cloned downstream of RTB by ligating the *Xho*I site on the 3' extreme of RTB to the *Sal*I site at the 5' of the p24 sequence (Figure 1B). The RTB/p24 fusion was cloned into the flanking sites *Eco*RI and *Hind*III of the *E. coli *expression vector pTrcHisA, which directs gene expression with the *trc *(*trc-lac*) promoter. In addition, p24 alone was also cloned in the vector pTrcHisA, at the *Xho*I and *Hind*III sites (Figure 1A). Both genes inserted into the expression vector were sequenced to confirm in-frame cloning. The pTrcHis vector contains a histidine tag employed for affinity chromatography purification.

### E. coli expression of RTB/p24 and p24 HIV proteins

Six *E. coli *strains were tested to obtain the best levels of expression and solubility of the RTB/p24 and p24 proteins in cytosolic fractions: HMS 174, HMS Rosetta 2, HMS LysE, BL21 Rosetta 2, Rosetta-gami and RP2. Two temperatures, 21°C and 37°C, were also tested accordingly. A single colony harboring the plasmid was inoculated on 25 ml of LB media in the presence of ampicilin (100 mg/l), and cultivated overnight at 37°C. The culture was transferred to 250 ml of LB medium and incubated to an OD600 of 0.5–0.7; after that, recombinant proteins synthesis was induced with IPTG (0.5 mM) during 5 to 7 h. Cells were harvested by centrifugation at 4000 rpm for 15 minutes. Cellular fractions of pellet and supernatant were diluted in Laemmli loading buffer, boiled at 95°C for 5 min and analyzed by SDS-PAGE and Coomassie blue staining.

### Purification of recombinant proteins

After recombinant protein induction, cellular pellets were resuspended in lysis buffer. We tested four different concentrations of NaCl (50, 75, 150 and 300 mM) and four pH conditions (pH 6, 7, 8 and 9), in the lysis buffer to obtain optimal solubilization of the recombinant RTB/p24 protein during affinity chromatography. Thus, cellular pellets were resuspended in the standarized lysis buffer (50 mM TrisHCl pH 7.0, 300 mM NaCl, 20 mM Imidazole, 1 mM PMSF, 1 mg/ml lysozyme). Protein extracts were incubated for 30 min at 4°C. Lysis was performed by the freeze-thaw lysozyme procedure as described previously [17]. Following centrifugation at 12000 rpm for 30 min, protein soluble fractions were filtered using a 0.4 μM Millipore filter and immediately passed through a Nickel-sepharose column (General Electric, US). Columns were pre-equilibrated with binding buffer (50 mM TrisHCl, 300 mM NaCl and 20 mM Imidazole pH 7.0). Extensive washes with binding buffer were applied to remove nonspecific protein interactions using the previously described binding buffer supplemented with 40 mM Imidazole. His-tagged recombinant proteins were recovered by application of binding buffer containing 500 mM Imidazole. The recovered fractions were dialyzed in PBS pH 7.4 overnight.

### Protein analysis

Protein concentration was estimated by using the Bradford reagent (Sigma). Recombinant proteins were subjected to western blot for immuno-detection. Consequently, proteins were transferred from the SDS gel (10%) on to a PVDF membrane (Amersham) and probed with antibodies, after overnight incubation with skim milk at 5% in TTBS buffer (100 mM TrisHCl pH 7.5, 150 mM NaCl, and 0.05% Tween-20). Mouse anti-HIS antibodies (Roche) were diluted in TTBS at 1/1000 and hybridized with the membrane for 1 h, at room temperature. Also, rabbit anti-Ricin antibodies (Sigma) at 1/3000 and mouse anti-p24 antibodies (Millipore) at 1/1000 dilutions were used for detection of recombinant proteins. After incubation, the membrane was washed three times for 10 min each and the secondary antibodies, diluted to 1/10,000, were added and incubated for an additional h at room temperature.

### Evaluation of biological activity of RTB/p24 in vitro

Interaction between the glycoprotein asialofetuin and RTB/p24 chimeric protein was analyzed by binding ELISA. Microtiter plates (Costar) were coated with 20 μg per well of asialofetuin (Sigma) in bicarbonate buffer (15 mM Na_2_CO_3_, 35 mM NaHCO_3_, pH 9.6). Binding was done overnight at 4°C. After blocking with PBS-5% skim milk (PBSM) for 2 h at room temperature, known quantities of purified RTB/p24 and p24 proteins (1 μg, 0.5 μg, and 0.125 μg) were added to the plates in triplicate and incubated overnight at 4°C. Plates were washed with PBS-0.05% Tween-20 (PBST) and, after adding the anti-p24 monoclonal antibody at 1/500 in PBSM, they were incubated at 37°C for 2 h. Following three washes with PBST, anti-mouse HRP conjugated secondary antibody was added at 1/2500 in PBSM and the plates were incubated at 37°C for 2 h. Following 3 washes with PBST, plates were coated with 100 μl of peroxidase TMB substrate buffer (Sigma), incubated for 15 min at room temperature and the reaction stopped with 50μl of 1 N H_2_SO_4_, and read at 450 nm. An additional experiment to evaluate interaction between RTB/p24 and asialofetuin was performed. We prepared a sepharose column for affinity chromatography with immobilized asialofetuin. Five mg of asialofetuin were diluted in 0.1 M NaHCO_3 _and 0.5 M NaCl buffer, pH 8.3 and the solution was employed for immobilization of asialofetuin in Cyanogen bromide-activated-sepharose (Sigma), which (1 g) had been hydrated and washed with 200 ml of 1 mM HCl. Following blocking with 0.2 M glycine pH 8.0 overnight, the resin was extensively washed with 0.1 M sodium acetate/0.5 M NaCl pH 4.0 and with 0.1 M Tris HCl/0.5 M NaCl pH 8.0, in five intervals. Subsequently, the resin was loaded onto the sepharose column. About 25 μg of recombinant, purified RTB/p24 in 20 ml of PBS were loaded on to a column containing the sepharose with the immobilized asialofetuin. The column was washed with eight ml of buffer A (50 mM TrisHCl, 5 mM EDTA, 150 mM NaCl, and 0.1% Tween-20 pH 7.8). Following a wash with 10 ml of buffer B (50 mM TrisHCl, 5 mM EDTA, pH 7.8), proteins were eluted in 3 fractions of 1 ml each using elution buffer (100 mM Glycine pH 4.0). Fractions were collected on tubes containing a neutralization solution buffer (200 mM Tris HCl, pH 9.0).

### Immunization of mice

Groups of six female 6–8 week-old BALB/c mice were inoculated intraperitoneally (i.p.) or intranasally (i.n.) with purified RTB/p24 and p24 recombinant proteins in PBS. Mice were immunized i.p. in a volume of 200 μl, with the following doses per group: PBS, 15 μg of p24, 30 μg of RTB/p24, 15 μg of p24 plus complete Freund's adjuvant (CFA), and 30 μg of RTB/p24 with CFA. Mice were boosted on days 15 and 30 using the same protocol, except that incomplete Freund's adjuvant (IFA) was used instead of CFA. We used 30 μg of the fusion protein RTB/p24 since RTB and p24 are present in equimolar proportion in the chimeric protein. For i.n. immunization, groups of six BALB/c mice were anesthetized with 25 μg of sodium phenobarbital in 0.2 ml of PBS per mouse, administered intraperitoneally. Recombinant protein doses were administered in a volume of 30 μl of PBS, as follows: PBS containing 5 μg of CT adjuvant (Sigma), 15 μg of p24, 30 μg of RTB/p24, 15 μg of p24 mixed with 5 μg of CT and, 30 μg of RTB/p24 mixed with 5 μg of CT adjuvant. Intranasal immunizations were performed using the same protocol as that for i.p. immunizations. All mice were bled before each inoculation and 15 days after the last immunization. Blood samples were kept at 4°C for 2 h, and serum was obtained by centrifugation at 4000 rpm for 10 min, at 4°C.

### Determination of mice Ab levels to RTB/p24 and p24 recombinant proteins

The levels of IgG antibodies anti-p24 were analyzed by ELISA during the time course of immunizations. A preliminary ELISA was performed to determine the optimal concentration of the purified p24 antigen and the best mice serum dilutions. We prepared serial dilutions, ranging from 1 μg to 0.00781 μg in triplicate wells and the protein was detected using three dilutions of the commercial monoclonal mouse anti-p24 antibody (1/500, 1/1000 and 1/2000). Data of p24 concentration were plotted against mice serum dilutions (data not show). From this experiment, we decided to employ 0.2 μg of p24 and to test mice serum samples at a dilution of 1/200 in subsequent experiments. Microtiter plates were coated with 200 ng/well of purified p24 in 50 μl of PBS and incubated at 4°C overnight. Following three washes with 150 μl/well of PBST, wells were blocked with 200 μl/well of PBSM for 2 h at room temperature. Plates were washed with PBST before addition of 50 μl/well of the serum samples at a dilution of 1/200 in PBSM. Plates were incubated at 37°C for 1 h, the wells washed extensively with PBST and 50 μl of HRP-conjugated, anti-mouse IgG secondary antibody diluted at 1/2500 in PBSM added to the wells. Four final washes with PBSM were given prior to addition of 50 μl/well OPD peroxidase substrate (Sigma). After incubation at room temperature for 8–10 min color development was read at 492 nm in an ELISA plate reader (Multiskan, Labsystem). The levels of anti-p24 antibody were determined in each serum sample and the values were used for estimation of standard deviations. All serum samples were analyzed in triplicate and the results were plotted to determine the anti-p24 antibody levels.

## Competing interests

The authors declare that they have no competing interests.

## Authors' contributions

AJDT carried out the vector construction, purification of the proteins and *in vitro *biological studies. EES, MLGX and AJDT performed the immunological assays in mice. FREG designed the immunological assays, participated in discussion of results and revision of the manuscript. MAGL conceived of the study, participated in its design and coordination and wrote the manuscript. All authors read and approved the final manuscript.
